# 4-(5,3′-Dimethyl-5′-oxo-2-phenyl-2′,5′-dihydro-2*H*-[3,4′]bipyrazol-1′-yl)benzene­sulfonamide monohydrate

**DOI:** 10.1107/S1600536811034453

**Published:** 2011-08-27

**Authors:** Abdullah M. Asiri, Abdulrahman O. Al-Youbi, Hassan M. Faidallah, Khalid A. Alamry, Seik Weng Ng

**Affiliations:** a Chemistry Department, Faculty of Science, King Abdulaziz University, PO Box 80203 Jeddah, Saudi Arabia; b Center of Excellence for Advanced Materials Research, King Abdulaziz University, PO Box 80203 Jeddah, Saudi Arabia; c Department of Chemistry, University of Malaya, 50603 Kuala Lumpur, Malaysia

## Abstract

In the title compound, C_20_H_19_N_5_O_3_S·H_2_O, the pyrazole ring is connected to a pyrazolone ring, and the two five-membered rings are aligned at 45.0 (1)°. The pyrazole ring is connected to a phenyl ring and the two are twisted by 42.7 (1)°. Finally, the pyrazolone ring is connected to a benzene ring and the two are twisted by 19.5 (1)°. The N—H and –NH_2_ portions and the solvent water mol­ecules are engaged in N—H⋯N, N—H⋯O and O—H⋯O hydrogen-bonding inter­actions to generate a three-dimensional network.

## Related literature

For related pyrazole–benzene­sulfonamides, see: Al-Youbi *et al.* (2011 ▶); Asiri *et al.* (2011 ▶).
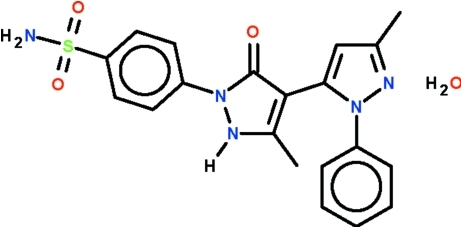


## Experimental

### 

#### Crystal data

C_20_H_19_N_5_O_3_S·H_2_O
*M* *_r_* = 427.48
Monoclinic, 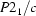

*a* = 11.1570 (5) Å
*b* = 12.3305 (5) Å
*c* = 14.9228 (5) Å
β = 107.142 (4)°
*V* = 1961.75 (14) Å^3^
*Z* = 4
Mo *K*α radiation
μ = 0.21 mm^−1^
*T* = 100 K
0.30 × 0.25 × 0.20 mm

#### Data collection

Agilent SuperNova Dual diffractometer with Atlas detector
Absorption correction: multi-scan (*CrysAlis PRO*; Agilent, 2010 ▶) *T*_min_ = 0.941, *T*_max_ = 0.960
9403 measured reflections
4382 independent reflections
3259 reflections with *I* > 2σ(*I*)
*R*_int_ = 0.039

#### Refinement

*R*[*F* ^2^ > 2σ(*F* ^2^)] = 0.048
*wR*(*F* ^2^) = 0.130
*S* = 1.01
4382 reflections
288 parameters
5 restraints
H atoms treated by a mixture of independent and constrained refinement
Δρ_max_ = 0.51 e Å^−3^
Δρ_min_ = −0.54 e Å^−3^

### 

Data collection: *CrysAlis PRO* (Agilent, 2010 ▶); cell refinement: *CrysAlis PRO*; data reduction: *CrysAlis PRO*; program(s) used to solve structure: *SHELXS97* (Sheldrick, 2008 ▶); program(s) used to refine structure: *SHELXL97* (Sheldrick, 2008 ▶); molecular graphics: *X-SEED* (Barbour, 2001 ▶); software used to prepare material for publication: *publCIF* (Westrip, 2010 ▶).

## Supplementary Material

Crystal structure: contains datablock(s) global, I. DOI: 10.1107/S1600536811034453/xu5305sup1.cif

Structure factors: contains datablock(s) I. DOI: 10.1107/S1600536811034453/xu5305Isup2.hkl

Supplementary material file. DOI: 10.1107/S1600536811034453/xu5305Isup3.cml

Additional supplementary materials:  crystallographic information; 3D view; checkCIF report

## Figures and Tables

**Table 1:** Hydrogen-bond geometry (Å, °)

*D*—H⋯*A*	*D*—H	H⋯*A*	*D*⋯*A*	*D*—H⋯*A*
N3—H3⋯N2^i^	0.88 (1)	2.05 (1)	2.927 (3)	175 (2)
N5—H51⋯O1^i^	0.88 (1)	2.05 (1)	2.913 (3)	165 (2)
N5—H52⋯O1*W*^ii^	0.88 (1)	2.09 (1)	2.932 (3)	161 (2)
O1*W*—H11⋯O1	0.84 (1)	1.94 (1)	2.769 (2)	169 (3)
O1*W*—H12⋯O2^iii^	0.84 (1)	2.38 (2)	3.158 (2)	154 (3)

Symmetry codes: (i) 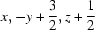; (ii) 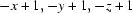; (iii) 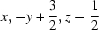.

**Fig. 1.**
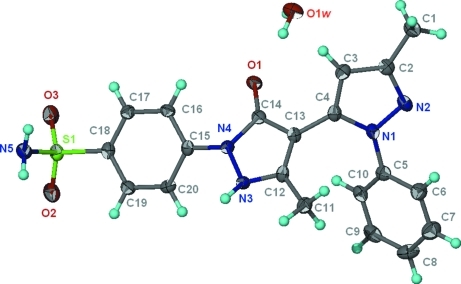
Thermal ellipsoid plot (Barbour, 2001) of C20H21N5O4S at the 70% probability level; H atoms are drawn as spheres of arbitrary radius. The molecule lies on a center-of-inversion.

## supplementary crystallographic information

### Comment

This study extends on structural studies (Asiri *et al.*, 2011; Al-Youbi *et al.*, 2011) of compounds having a pyrrole ring as well as a benzesulfonamide unit, the combination of which is expected to lead to enhanced medicinal activity. The present compound is a pyrazole that is connected to a pyrazorolone (Scheme I, Fig. 1). The two five-membered ring-systems are aligned at 45.0 (1)°. The pyrazole ring-system is connected to a phenyl ring and the two are twisted by 42.7 (1)°; the pyrazolone ring-system is connected to a benzene ring and the two are twisted by 19.5 (1)°. The ═N—H and –NH_2_ portions and the lattice water molecule are engagd in N—H···N, N—H···O and O—H···O hydrogen bonding interactions to generate a three-dimensional network (Table 1). The amino N atom shows pyramidal coordination.

### Experimental

4-Acetoacetyl-3-methyl-1-(*p*-sulpfamylphenyl)-2-pyrazolin-5-one (0.05 mol) and phenylhydrazine (0.05 mol) were heated in a mixture of ethanol (50 ml) and acetic acid (50 ml) for 2 h. The mixture was allowed and the solid was collected and recrystallized from ethanol to give colorless prisms; m.p. 578–579 K.

### Refinement

Carbon-bound H atoms were placed in calculated positions [C—H 0.95–0.98 Å, *U*_iso_(H) 1.2–1.5*U*_eq_(C)] and were included in the refinement in the riding model approximation. The water and amino H atoms were located in a difference Fourier map, and were refined with distance restraints of O—H 0.84 (1) Å and N—H 0.88 (1) Å; their temperature factors were refined.

### Crystal data

**Table:**

C_20_H_19_N_5_O_3_S·H_2_O	*F*(000) = 896
*M**_r_* = 427.48	*D*_x_ = 1.447 Mg m^−^^3^
Monoclinic, *P*2_1_/*c*	Mo *K*α radiation, λ = 0.71073 Å
Hall symbol: -P 2ybc	Cell parameters from 3286 reflections
*a* = 11.1570 (5) Å	θ = 2.5–29.4°
*b* = 12.3305 (5) Å	µ = 0.21 mm^−^^1^
*c* = 14.9228 (5) Å	*T* = 100 K
β = 107.142 (4)°	Prism, colourless
*V* = 1961.75 (14) Å^3^	0.30 × 0.25 × 0.20 mm
*Z* = 4	

### Data collection

**Table:**

Agilent SuperNova Dual diffractometer with Atlas detector	4382 independent reflections
Radiation source: SuperNova (Mo) X-ray Source	3259 reflections with *I* > 2σ(*I*)
mirror	*R*_int_ = 0.039
Detector resolution: 10.4041 pixels mm^-1^	θ_max_ = 27.5°, θ_min_ = 2.5°
ω scans	*h* = −11→14
Absorption correction: multi-scan (*CrysAlis PRO*; Agilent, 2010)	*k* = −13→15
*T*_min_ = 0.941, *T*_max_ = 0.960	*l* = −19→19
9403 measured reflections	

### Refinement

**Table:**

Refinement on *F*^2^	Primary atom site location: structure-invariant direct methods
Least-squares matrix: full	Secondary atom site location: difference Fourier map
*R*[*F*^2^ > 2σ(*F*^2^)] = 0.048	Hydrogen site location: inferred from neighbouring sites
*wR*(*F*^2^) = 0.130	H atoms treated by a mixture of independent and constrained refinement
*S* = 1.01	*w* = 1/[σ^2^(*F*_o_^2^) + (0.0564*P*)^2^ + 0.8598*P*] where *P* = (*F*_o_^2^ + 2*F*_c_^2^)/3
4382 reflections	(Δ/σ)_max_ = 0.001
288 parameters	Δρ_max_ = 0.51 e Å^−^^3^
5 restraints	Δρ_min_ = −0.54 e Å^−^^3^

### Fractional atomic coordinates and isotropic or equivalent isotropic displacement parameters (Å^2^)

**Table:**

	*x*	*y*	*z*	*U*_iso_*/*U*_eq_	
S1	0.71034 (5)	0.80796 (5)	0.74400 (3)	0.01854 (16)	
O1	0.42087 (15)	0.58177 (12)	0.31140 (10)	0.0197 (4)	
O2	0.73924 (16)	0.92176 (13)	0.75124 (10)	0.0249 (4)	
O3	0.80889 (15)	0.73007 (14)	0.75709 (10)	0.0257 (4)	
O1W	0.51292 (17)	0.41557 (14)	0.22561 (12)	0.0262 (4)	
H11	0.476 (2)	0.4640 (17)	0.2472 (18)	0.039*	
H12	0.5866 (13)	0.439 (2)	0.2367 (19)	0.039*	
N1	0.17489 (17)	0.67063 (15)	0.04860 (11)	0.0166 (4)	
N2	0.15213 (17)	0.59426 (15)	−0.02130 (12)	0.0187 (4)	
N3	0.24829 (17)	0.80650 (15)	0.33546 (12)	0.0163 (4)	
H3	0.217 (2)	0.8392 (17)	0.3758 (13)	0.020*	
N4	0.34314 (17)	0.73153 (15)	0.37024 (11)	0.0161 (4)	
N5	0.63792 (19)	0.78264 (16)	0.82054 (13)	0.0209 (4)	
H51	0.5824 (19)	0.8327 (16)	0.8217 (17)	0.025*	
H52	0.609 (2)	0.7161 (11)	0.8133 (17)	0.025*	
C1	0.1501 (2)	0.39583 (19)	−0.03171 (15)	0.0230 (5)	
H1A	0.1414	0.4119	−0.0976	0.035*	
H1B	0.0744	0.3588	−0.0271	0.035*	
H1C	0.2231	0.3489	−0.0063	0.035*	
C2	0.1673 (2)	0.49882 (18)	0.02272 (14)	0.0189 (5)	
C3	0.1981 (2)	0.51292 (19)	0.11991 (14)	0.0188 (5)	
H3A	0.2127	0.4575	0.1661	0.023*	
C4	0.2029 (2)	0.62324 (18)	0.13530 (14)	0.0170 (5)	
C5	0.1673 (2)	0.78201 (18)	0.02104 (14)	0.0176 (5)	
C6	0.0699 (2)	0.81337 (19)	−0.05719 (15)	0.0220 (5)	
H6	0.0077	0.7625	−0.0885	0.026*	
C7	0.0647 (2)	0.9183 (2)	−0.08844 (17)	0.0293 (6)	
H7	−0.0005	0.9399	−0.1424	0.035*	
C8	0.1543 (2)	0.9932 (2)	−0.04149 (17)	0.0280 (6)	
H8	0.1499	1.0660	−0.0630	0.034*	
C9	0.2497 (2)	0.9619 (2)	0.03634 (16)	0.0241 (5)	
H9	0.3101	1.0136	0.0688	0.029*	
C10	0.2580 (2)	0.85513 (18)	0.06757 (14)	0.0193 (5)	
H10	0.3250	0.8329	0.1200	0.023*	
C11	0.0624 (2)	0.83177 (19)	0.20154 (15)	0.0220 (5)	
H11A	0.0022	0.7821	0.1604	0.033*	
H11B	0.0820	0.8907	0.1641	0.033*	
H11C	0.0261	0.8621	0.2484	0.033*	
C12	0.1786 (2)	0.77221 (19)	0.24935 (13)	0.0173 (5)	
C13	0.2349 (2)	0.68339 (18)	0.22385 (14)	0.0160 (5)	
C14	0.3403 (2)	0.65484 (18)	0.30161 (14)	0.0164 (5)	
C15	0.4352 (2)	0.74994 (18)	0.45697 (13)	0.0157 (5)	
C16	0.5117 (2)	0.66528 (18)	0.50240 (14)	0.0167 (5)	
H16	0.5053	0.5957	0.4740	0.020*	
C17	0.5975 (2)	0.68359 (18)	0.58972 (14)	0.0178 (5)	
H17	0.6511	0.6267	0.6211	0.021*	
C18	0.6048 (2)	0.78517 (18)	0.63096 (14)	0.0172 (5)	
C19	0.5284 (2)	0.86945 (18)	0.58551 (14)	0.0178 (5)	
H19	0.5337	0.9386	0.6145	0.021*	
C20	0.4444 (2)	0.85223 (18)	0.49786 (14)	0.0176 (5)	
H20	0.3932	0.9100	0.4657	0.021*	

### Atomic displacement parameters (Å^2^)

**Table:**

	*U*^11^	*U*^22^	*U*^33^	*U*^12^	*U*^13^	*U*^23^
S1	0.0154 (3)	0.0224 (3)	0.0164 (3)	−0.0013 (2)	0.0024 (2)	−0.0019 (2)
O1	0.0191 (9)	0.0182 (8)	0.0203 (7)	0.0040 (7)	0.0035 (6)	−0.0034 (6)
O2	0.0256 (10)	0.0252 (9)	0.0226 (8)	−0.0094 (7)	0.0052 (7)	−0.0042 (7)
O3	0.0166 (9)	0.0354 (10)	0.0219 (8)	0.0055 (7)	0.0008 (6)	−0.0032 (7)
O1W	0.0283 (10)	0.0204 (9)	0.0329 (9)	−0.0007 (8)	0.0136 (8)	−0.0039 (7)
N1	0.0164 (10)	0.0189 (10)	0.0141 (8)	−0.0023 (8)	0.0039 (7)	−0.0021 (7)
N2	0.0166 (10)	0.0205 (10)	0.0193 (9)	−0.0032 (8)	0.0060 (7)	−0.0062 (8)
N3	0.0176 (10)	0.0160 (10)	0.0156 (8)	0.0046 (8)	0.0054 (7)	0.0000 (7)
N4	0.0158 (10)	0.0163 (9)	0.0152 (8)	0.0036 (8)	0.0033 (7)	0.0001 (7)
N5	0.0233 (11)	0.0198 (10)	0.0197 (9)	−0.0011 (9)	0.0063 (8)	−0.0022 (9)
C1	0.0239 (13)	0.0216 (12)	0.0235 (11)	−0.0024 (10)	0.0069 (9)	−0.0030 (10)
C2	0.0160 (11)	0.0198 (12)	0.0206 (10)	−0.0025 (9)	0.0050 (8)	−0.0009 (9)
C3	0.0169 (11)	0.0201 (12)	0.0188 (10)	0.0000 (9)	0.0045 (8)	0.0023 (9)
C4	0.0127 (11)	0.0219 (12)	0.0156 (10)	−0.0006 (9)	0.0030 (8)	−0.0006 (9)
C5	0.0182 (12)	0.0181 (11)	0.0185 (10)	−0.0005 (9)	0.0083 (9)	−0.0004 (9)
C6	0.0209 (13)	0.0229 (13)	0.0215 (11)	−0.0013 (10)	0.0052 (9)	0.0004 (10)
C7	0.0255 (14)	0.0292 (14)	0.0303 (12)	0.0041 (11)	0.0038 (10)	0.0039 (11)
C8	0.0280 (14)	0.0181 (12)	0.0399 (14)	0.0025 (11)	0.0131 (11)	0.0051 (11)
C9	0.0254 (13)	0.0227 (13)	0.0272 (12)	−0.0071 (10)	0.0124 (10)	−0.0081 (10)
C10	0.0211 (12)	0.0211 (12)	0.0172 (10)	−0.0011 (10)	0.0080 (9)	−0.0036 (9)
C11	0.0210 (13)	0.0243 (13)	0.0186 (10)	0.0051 (10)	0.0026 (9)	0.0012 (10)
C12	0.0172 (12)	0.0220 (12)	0.0132 (9)	−0.0010 (9)	0.0055 (8)	0.0019 (9)
C13	0.0139 (11)	0.0188 (11)	0.0153 (9)	−0.0010 (9)	0.0044 (8)	−0.0009 (9)
C14	0.0198 (12)	0.0148 (11)	0.0164 (10)	−0.0021 (9)	0.0080 (9)	0.0005 (9)
C15	0.0158 (11)	0.0189 (12)	0.0134 (9)	−0.0008 (9)	0.0058 (8)	0.0006 (9)
C16	0.0191 (12)	0.0142 (11)	0.0181 (10)	0.0017 (9)	0.0076 (8)	−0.0012 (9)
C17	0.0179 (12)	0.0184 (12)	0.0169 (10)	0.0033 (9)	0.0051 (8)	0.0012 (9)
C18	0.0145 (11)	0.0221 (12)	0.0147 (9)	−0.0023 (9)	0.0040 (8)	0.0003 (9)
C19	0.0211 (12)	0.0160 (11)	0.0173 (10)	−0.0007 (9)	0.0072 (9)	−0.0014 (9)
C20	0.0183 (12)	0.0150 (11)	0.0198 (10)	0.0017 (9)	0.0061 (9)	0.0021 (9)

### Geometric parameters (Å, °)

**Table:**

S1—O3	1.4293 (17)	C5—C6	1.395 (3)
S1—O2	1.4366 (16)	C6—C7	1.371 (3)
S1—N5	1.612 (2)	C6—H6	0.9500
S1—C18	1.772 (2)	C7—C8	1.390 (3)
O1—C14	1.251 (3)	C7—H7	0.9500
O1W—H11	0.844 (10)	C8—C9	1.379 (3)
O1W—H12	0.840 (10)	C8—H8	0.9500
N1—C4	1.369 (3)	C9—C10	1.391 (3)
N1—N2	1.372 (2)	C9—H9	0.9500
N1—C5	1.429 (3)	C10—H10	0.9500
N2—C2	1.334 (3)	C11—C12	1.477 (3)
N3—C12	1.359 (3)	C11—H11A	0.9800
N3—N4	1.386 (2)	C11—H11B	0.9800
N3—H3	0.880 (9)	C11—H11C	0.9800
N4—C14	1.387 (3)	C12—C13	1.371 (3)
N4—C15	1.413 (3)	C13—C14	1.431 (3)
N5—H51	0.878 (10)	C15—C20	1.392 (3)
N5—H52	0.876 (10)	C15—C16	1.392 (3)
C1—C2	1.489 (3)	C16—C17	1.390 (3)
C1—H1A	0.9800	C16—H16	0.9500
C1—H1B	0.9800	C17—C18	1.387 (3)
C1—H1C	0.9800	C17—H17	0.9500
C2—C3	1.399 (3)	C18—C19	1.388 (3)
C3—C4	1.378 (3)	C19—C20	1.382 (3)
C3—H3A	0.9500	C19—H19	0.9500
C4—C13	1.465 (3)	C20—H20	0.9500
C5—C10	1.381 (3)		
			
O3—S1—O2	119.96 (11)	C8—C7—H7	119.9
O3—S1—N5	107.40 (10)	C9—C8—C7	120.1 (2)
O2—S1—N5	106.50 (10)	C9—C8—H8	120.0
O3—S1—C18	106.98 (10)	C7—C8—H8	120.0
O2—S1—C18	107.33 (10)	C8—C9—C10	120.3 (2)
N5—S1—C18	108.23 (10)	C8—C9—H9	119.8
H11—O1W—H12	104 (3)	C10—C9—H9	119.8
C4—N1—N2	111.40 (18)	C5—C10—C9	118.9 (2)
C4—N1—C5	131.29 (18)	C5—C10—H10	120.5
N2—N1—C5	117.31 (16)	C9—C10—H10	120.5
C2—N2—N1	105.24 (16)	C12—C11—H11A	109.5
C12—N3—N4	107.92 (17)	C12—C11—H11B	109.5
C12—N3—H3	123.8 (16)	H11A—C11—H11B	109.5
N4—N3—H3	117.7 (15)	C12—C11—H11C	109.5
N3—N4—C14	109.23 (16)	H11A—C11—H11C	109.5
N3—N4—C15	120.29 (17)	H11B—C11—H11C	109.5
C14—N4—C15	129.64 (19)	N3—C12—C13	109.25 (19)
S1—N5—H51	112.5 (16)	N3—C12—C11	118.57 (19)
S1—N5—H52	109.6 (17)	C13—C12—C11	132.18 (19)
H51—N5—H52	115 (3)	C12—C13—C14	107.79 (18)
C2—C1—H1A	109.5	C12—C13—C4	130.18 (19)
C2—C1—H1B	109.5	C14—C13—C4	122.0 (2)
H1A—C1—H1B	109.5	O1—C14—N4	123.05 (19)
C2—C1—H1C	109.5	O1—C14—C13	131.5 (2)
H1A—C1—H1C	109.5	N4—C14—C13	105.40 (19)
H1B—C1—H1C	109.5	C20—C15—C16	120.71 (19)
N2—C2—C3	110.95 (19)	C20—C15—N4	119.14 (19)
N2—C2—C1	120.43 (19)	C16—C15—N4	120.09 (19)
C3—C2—C1	128.6 (2)	C17—C16—C15	119.3 (2)
C4—C3—C2	106.32 (19)	C17—C16—H16	120.4
C4—C3—H3A	126.8	C15—C16—H16	120.4
C2—C3—H3A	126.8	C18—C17—C16	119.8 (2)
N1—C4—C3	106.09 (18)	C18—C17—H17	120.1
N1—C4—C13	124.3 (2)	C16—C17—H17	120.1
C3—C4—C13	129.60 (19)	C17—C18—C19	120.79 (19)
C10—C5—C6	121.0 (2)	C17—C18—S1	120.08 (17)
C10—C5—N1	120.7 (2)	C19—C18—S1	119.12 (17)
C6—C5—N1	118.2 (2)	C20—C19—C18	119.7 (2)
C7—C6—C5	119.4 (2)	C20—C19—H19	120.2
C7—C6—H6	120.3	C18—C19—H19	120.2
C5—C6—H6	120.3	C19—C20—C15	119.7 (2)
C6—C7—C8	120.2 (2)	C19—C20—H20	120.1
C6—C7—H7	119.9	C15—C20—H20	120.1
			
C4—N1—N2—C2	−0.5 (2)	N1—C4—C13—C12	46.1 (4)
C5—N1—N2—C2	178.93 (19)	C3—C4—C13—C12	−136.2 (3)
C12—N3—N4—C14	5.7 (2)	N1—C4—C13—C14	−132.9 (2)
C12—N3—N4—C15	176.18 (18)	C3—C4—C13—C14	44.9 (4)
N1—N2—C2—C3	0.7 (2)	N3—N4—C14—O1	174.80 (19)
N1—N2—C2—C1	−179.8 (2)	C15—N4—C14—O1	5.5 (4)
N2—C2—C3—C4	−0.6 (3)	N3—N4—C14—C13	−2.6 (2)
C1—C2—C3—C4	180.0 (2)	C15—N4—C14—C13	−171.9 (2)
N2—N1—C4—C3	0.1 (2)	C12—C13—C14—O1	−178.5 (2)
C5—N1—C4—C3	−179.2 (2)	C4—C13—C14—O1	0.6 (4)
N2—N1—C4—C13	178.32 (19)	C12—C13—C14—N4	−1.4 (2)
C5—N1—C4—C13	−1.0 (4)	C4—C13—C14—N4	177.73 (19)
C2—C3—C4—N1	0.3 (2)	N3—N4—C15—C20	−12.5 (3)
C2—C3—C4—C13	−177.8 (2)	C14—N4—C15—C20	155.8 (2)
C4—N1—C5—C10	44.3 (3)	N3—N4—C15—C16	164.83 (19)
N2—N1—C5—C10	−135.0 (2)	C14—N4—C15—C16	−26.9 (3)
C4—N1—C5—C6	−139.3 (2)	C20—C15—C16—C17	0.3 (3)
N2—N1—C5—C6	41.4 (3)	N4—C15—C16—C17	−176.93 (19)
C10—C5—C6—C7	0.3 (3)	C15—C16—C17—C18	0.8 (3)
N1—C5—C6—C7	−176.2 (2)	C16—C17—C18—C19	−0.8 (3)
C5—C6—C7—C8	−1.2 (4)	C16—C17—C18—S1	177.88 (17)
C6—C7—C8—C9	0.6 (4)	O3—S1—C18—C17	25.0 (2)
C7—C8—C9—C10	0.9 (4)	O2—S1—C18—C17	154.97 (18)
C6—C5—C10—C9	1.3 (3)	N5—S1—C18—C17	−90.4 (2)
N1—C5—C10—C9	177.60 (19)	O3—S1—C18—C19	−156.30 (18)
C8—C9—C10—C5	−1.9 (3)	O2—S1—C18—C19	−26.3 (2)
N4—N3—C12—C13	−6.7 (2)	N5—S1—C18—C19	88.25 (19)
N4—N3—C12—C11	173.17 (19)	C17—C18—C19—C20	−0.4 (3)
N3—C12—C13—C14	5.0 (2)	S1—C18—C19—C20	−179.08 (17)
C11—C12—C13—C14	−174.8 (2)	C18—C19—C20—C15	1.5 (3)
N3—C12—C13—C4	−174.0 (2)	C16—C15—C20—C19	−1.5 (3)
C11—C12—C13—C4	6.2 (4)	N4—C15—C20—C19	175.77 (19)

### Hydrogen-bond geometry (Å, °)

**Table:**

*D*—H···*A*	*D*—H	H···*A*	*D*···*A*	*D*—H···*A*
N3—H3···N2^i^	0.88 (1)	2.05 (1)	2.927 (3)	175 (2)
N5—H51···O1^i^	0.88 (1)	2.05 (1)	2.913 (3)	165 (2)
N5—H52···O1W^ii^	0.88 (1)	2.09 (1)	2.932 (3)	161 (2)
O1W—H11···O1	0.84 (1)	1.94 (1)	2.769 (2)	169 (3)
O1W—H12···O2^iii^	0.84 (1)	2.38 (2)	3.158 (2)	154 (3)

Symmetry codes: (i) *x*, −*y*+3/2, *z*+1/2; (ii) −*x*+1, −*y*+1, −*z*+1; (iii) *x*, −*y*+3/2, *z*−1/2.
